# Summary of the best evidence for early rehabilitation in ICU patients receiving continuous renal replacement therapy

**DOI:** 10.3389/fmed.2026.1730618

**Published:** 2026-01-28

**Authors:** Yang Zou, Tingting Tang, Chunmei Wang, Jie Gao, Chunyan Wang

**Affiliations:** 1 Department of Critical Care Medicine, West China Hospital/West China School of Nursing, Sichuan University, Chengdu, Sichuan, China; 2 Department of Nursing, Air Force Medical University, Xian, Shanxi, China

**Keywords:** best evidence, CRRT, early rehabilitation, ICU, nursing

## Abstract

**Aims:**

To summarize the best evidence for early rehabilitation in intensive care unit (ICU) patients receiving continuous renal replacement therapy, both domestically and internationally, with the aim of providing a reference for clinical decision-making.

**Methods:**

A systematic search of expert consensus, guidelines, systematic reviews, randomized controlled trials, and other literature related to early rehabilitation in ICU patients undergoing continuous renal replacement therapy from BMJ Best Practice, UpToDate, PubMed, JBI Evidence-Based Healthcare Center, Cochrane Library, CINAHL, Elsevier Science Direct, Web of Science, CBM, China National Knowledge Infrastructure (CNKI), Wanfang, Weipu databases, WHO, International Guideline Collaboration Network, National Institute for Health and Care Excellence (NICE), Scotland Intercollegiate Guidelines Network, and the National Guidelines Clearinghouse website was performed. The search period was from database inception until June 30, 2025. Two researchers conducted a quality assessment and synthesis of the included evidence.

**Results:**

A total of 8 studies were included in this research. These included 2 expert consensus papers, 2 systematic reviews, 1 quasi-experimental study, 1 group standard, 1 recommendation, and 1 evidence summary. The study summarizes the 20 best pieces of evidence for early rehabilitation in ICU patients undergoing continuous renal replacement therapy, covering five key areas: multidisciplinary collaboration, preactivity preparation and assessment, activity contraindications, exercise rehabilitation strategies, and monitoring during activity.

**Conclusion:**

This study summarizes the best evidence for early rehabilitation in ICU patients receiving continuous renal replacement therapy, providing an evidence-based foundation for healthcare professionals to formulate rehabilitation strategies. By tailoring interventions to the individual patient’s condition, personalized measures can be implemented to further enhance rehabilitation quality.

**Implications for the profession and patient care:**

This study provides reference evidence for clinical healthcare personnel to implement scientifically based and personalized early rehabilitation strategies for ICU patients undergoing continuous renal replacement therapy to promote their recovery.

**Reporting method:**

This evidence summary was developed in accordance with the evidence synthesis reporting guidelines established by the Fudan University Center for Evidence-based Nursing, which themselves derive from the methodological framework for evidence summarization promulgated by the Joanna Briggs Institute. This study follows the evidence summary report guidelines of the Evidence-Based Nursing Center at Fudan University, registered under the title “Evidence Summary for Early Rehabilitation in ICU-CRRT Patients,” with registration number “ES20246522.”

**Systematic review registration:**

Fudan University Evidence-Based Nursing Center: [http://ebn.nursing.fudan.edu.cn/home].

## Introduction

Kidney replacement therapy (KRT) is an important method for treating and salvaging patients with acute kidney injury (AKI). It can be divided into intermittent hemodialysis, continuous renal replacement therapy (CRRT), and peritoneal dialysis (PD). Intermittent hemodialysis is commonly used for hemodynamically stable patients, whereas CRRT and PD are often used for hemodynamically unstable patients (1). Continuous renal replacement therapy (CRRT) is a primary method for treating renal failure, sepsis, septic shock, septicemia, and multiple organ dysfunction syndrome (MODS) (2). Compared with intermittent hemodialysis, CRRT offers advantages in enhancing hemodynamic stability, providing greater solute control, and enabling superior fluid balance management (3). In addition, it has been shown to reduce dialysis dependence (4).

Studies have shown that approximately 13.5% of ICU patients undergo continuous renal replacement therapy (CRRT) (5). During treatment, owing to risks such as catheter dislodgement, hemodynamic instability, bleeding at the puncture site, infection, and interruptions in CRRT, patients are often confined to bed rest, resulting in insufficient physical activity and exercise. Prolonged bed rest and immobilization can lead to adverse outcomes such as muscle atrophy, increased risk of thrombosis, shortened filter lifespan, prolonged duration of mechanical ventilation, extended length of hospital stay, difficulty weaning, and increased mortality (6). In addition, prolonged bed rest renders patients in the intensive care unit more susceptible to ICU-acquired weakness, which severely compromises their ability to perform activities of daily living and contributes to unfavorable clinical outcomes (7).

With the growing emphasis on early mobilization in critical care rehabilitation, an increasing number of researchers have begun to explore early rehabilitation programs for ICU patients. Hodgson et al. (8), on the basis of the concept of early goal-directed mobilization (EGDM), conducted a multicenter randomized controlled trial in ICU patients receiving mechanical ventilation. The results demonstrated that EGDM can enhance patients’ mobility and improve their ability to perform activities of daily living. Qin et al. (9) developed an early comprehensive rehabilitation program and, through a randomized controlled trial, investigated the effect of early mobilization in preventing ICU-acquired weakness among mechanically ventilated patients. The results demonstrated improvements in patients’ self-care ability, prevention of ICU-acquired weakness, and promotion of overall recovery. However, current guidelines on early mobilization primarily target ICU patients in general rather than those undergoing CRRT, and the recommendations are extensive and not entirely consistent. Therefore, this study systematically searched for and evaluated the available evidence to summarize the best evidence for early rehabilitation in ICU patients receiving continuous renal replacement therapy, providing an evidence-based foundation for clinical practice and management to promote early patient recovery.

## Materials and methods

### Problem establishment

The evidence-based question was formulated via the PIPOST tool from the Evidence-Based Nursing Center at Fudan University (10). Population (P): ICU patients receiving continuous renal replacement therapy. Intervention (I): Early rehabilitation and early mobilization exercises. Professional (P): The evidence implementers are the relevant healthcare personnel. Outcome (O): Safety and feasibility of early mobilization, improvement in muscle strength and mobility, and reduction in complications such as thrombosis and catheter dislodgement. Setting (S): Intensive care unit (ICU). Types of evidence (T): Expert consensus, guidelines, standards, protocols, recommendations, statements, systematic reviews, evidence summaries, clinical decision support, meta-analyses, and randomized controlled trials.

### Search strategy

The search was conducted sequentially on the basis of the “6S” evidence model. The databases searched included BMJ Best Practice, UpToDate, PubMed, JBI Evidence-Based Healthcare Center, Cochrane Library, CINAHL, Elsevier Science Direct, Web of Science, CBM, China National Knowledge Infrastructure (CNKI), Wanfang Database, and Weipu Database. Websites: WHO, International Guideline Collaboration Network, National Institute for Health and Care Excellence (NICE), Scottish Intercollegiate Guidelines Network (SIGN), U. S. National Guideline Clearinghouse, Ontario Nursing, American Society of Nephrology, and Yimai Tong.

Search terms: early rehabilitation, MeSH terms: early ambulation; free-text terms: accelerated ambulation, early mobilization, early mobilization, early activity, early activity, early rehabilitation, early rehabilitation*, early exercise, early movement, physical activity. CRRT, MeSH terms: Continuous Renal Replacement Therapy; Free-text terms: CRRT, Continuous RRT*, Continuous Renal Replacement Procedure, Continuous Venovenous Hemodiafiltration*, CVVHDF, Continuous Venovenous Hemodiafiltration*, Slow Continuous Ultrafiltration*, SCUF Technique*, Continuous Venovenous Hemofiltration*, CVVH Technique*, Continuous arteriovenous Hemofiltration*, Continuous arteriovenous ultrafiltration*, CAVHD, Continuous Venovenous Hemodialysis*, CVVHD, Continuous Venovenous Hemodialysis*. Critically ill patients, MeSH terms: critical care nursing, critical illness, critical care, intensive care units; free-text terms: intensive care never*, critical illness, critically ill, ICU, intensive care, mechanically ventilated, mechanical ventilation, ventilation. Others: guidelines, practice guidelines, consensuses, routines, recommendations, summaries, evidence summaries, practice recommendations, meta-analyses (MeSH terms), systematic reviews, meta-analyses, and systematic reviews.

**Figure 1:**
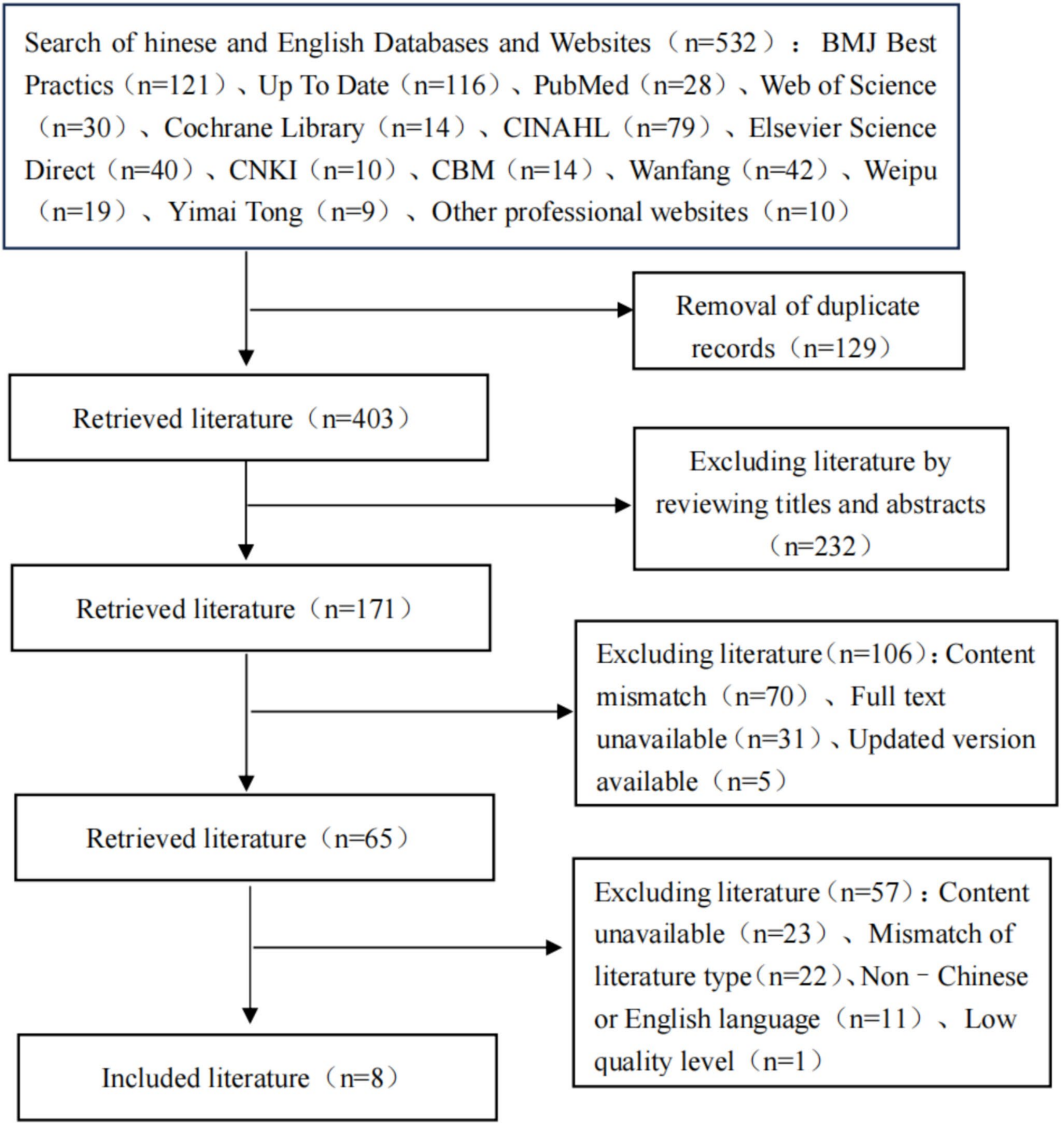
Literature screening flowchart.

For the Chinese databases, the search strategy used was as follows: (Topic: early rehabilitation) OR (Topic: early activity) OR (Topic: early exercise) OR (Topic:early movement) OR (Topic: early rehabilitation exercise) OR (Topic:early functional exercise) AND (Topic: ICU) OR (Topic: early care unit) OR (Topic: critical care ward) OR (Topic:critically ill patients) AND (Topic: CRRT) OR (Topic: continuous renal replacement therapy) OR (Topic: continuous blood purification) OR (Topic: ICU ward) OR (Topic: continuous venovenous hemofiltration) OR (Topic: continuous venovenous hemodiafiltration) OR (Topic: continuous venovenous filtration) OR (Topic: continuous venovenous hemodialysis) OR (Topic: slow continuous ultrafiltration). The search period spanned from database inception to June 30, 2025. The literature screening process is shown in Figure 1.

### Inclusion and exclusion criteria for evidence

The inclusion criteria were as follows: study population: ICU patients receiving continuous renal replacement therapy (CRRT); study content: early rehabilitation, early mobilization, or rehabilitation exercises; study types: expert consensus, guidelines, standards, protocols, recommendations, statements, systematic reviews, evidence summaries, clinical decision support, meta-analyses, or randomized controlled trials; and languages: Chinese or English. The exclusion criteria were as follows: full text unavailable or incomplete literature information; updated guidelines; and studies rated as low quality (Grade C) on the basis of a literature quality assessment.

### Quality evaluation of the literature

The AGREE II instrument (11) was used for the assessment of clinical guidelines and systematic reviews. For systematic reviews, expert consensus, recommendations, group standards, and quasiexperimental studies, the respective quality appraisal tools from the JBI Evidence-Based Healthcare Center (2016) were used. The quality of evidence summaries was assessed by tracing them back to the original studies and applying the appropriate appraisal criteria on the basis of the type of original research. Two researchers independently evaluated the literature, and in cases of disagreement or uncertainty, a third researcher made the final decision regarding inclusion or exclusion.

### Evidence synthesis and grading

The evidence was logically categorized according to different themes, and evidence with similar or complementary content was combined. For example, under team composition, the statements “Early mobilization programs are undertaken by all members of a multidisciplinary team (i.e., physical therapists, medical staff, and nurses), with clinicians holding ultimate decision-making responsibility” and “Early mobilization programs for CRRT patients are managed by the acute dialysis medical director, dialysis nurses, ICU nurses, critical care nursing specialists, and responsible nurses” were merged. Evidence with independent content was retained in its original form. When evidence from different sources was contradictory, the inclusion principle prioritized evidence-based sources, higher-quality evidence, and more recently published studies. After the evidence was synthesized, the 2014 edition of the JBI pre-grading system was used to grade the evidence, as shown in Table 1.

**Table 1:** JBI pre-grading system for evidence.

Level of evidence	Design types and descriptions
Level 1	This conclusion is primarily based on one or more well-designed randomized controlled trials
Level 2	his conclusion is primarily based on one or more well-designed quasi-experimental studies
Level 3	This conclusion is primarily based on observational-analytic studies or systematic reviews synthesizing multiple similar primary studies
Level 4	This conclusion is primarily based on observational-descriptive studies
Level 5	This conclusion is primarily based on expert opinion, basic research, pathophysiological principles, or consensus statements

The evidence was classified into levels 1–5 according to the different study design types, with level 1 being the highest and level 5 being the lowest (12, 13). The source and type of each evidence item were first identified, after which an initial level of evidence was assigned according to the JBI pre-grading table. This initial level was subsequently adjusted based on the quality and applicability of the evidence, taking into account the following factors: study limitations (whether the included studies had significant flaws in design, conduct, or analysis); consistency (whether findings were consistent across different studies); indirectness (whether the PICO elements—population, intervention, comparator, and outcome—corresponded to the clinical question under consideration, such as evidence derived from animal studies or different populations); imprecision (whether wide confidence intervals of effect estimates resulted in uncertainty of the findings); and publication bias (the likelihood of unpublished negative results). Evidence of high quality with no apparent limitations retained its initial level, whereas the presence of one or more serious issues led to downgrading of the evidence by one or more levels.

## Results

### Basic characteristics of the included studies

A total of 532 articles were retrieved for this study. After removing duplicates, screening titles and abstracts, reviewing full texts, and conducting quality assessments, eight studies were ultimately included (14–21). These included two expert consensus papers, two systematic reviews, one quasiexperimental study, one group standard, one recommendation, and one evidence summary. The basic information of the included studies is presented in Table 2.

**Table 2:** Basic information of the included studies.

Included studies	Year of publication	Source	Type of literature	The literature theme
Hodgson et al. (14)	2014	Pubmed	Expert consensus	Expert consensus and recommendations on the safety standards for mobilization of mechanically ventilated critically ill adult patients
Tang et al. (15)	2023	Yimai Tong	Expert consensus	Strategies for comprehensive management during the recovery phase of critically ill patients
Nydahl et al. (16)	2017	Pubmed	Systematic review	Safety analysis of mobilization and rehabilitation in ICU patients
Mayer et al. (17)	2020	Web of science	Systematic review	Analysis of the safety and feasibility of rehabilitation exercises and early mobilization in patients receiving continuous renal replacement therapy
Cheryl et al. (18)	2013	Pubmed	Quasiexperimental study	Safety and feasibility of early mobilization in CRRT patients
Chinese Nursing Association (19)	2023	Chinese Nursing Society Website	Group standard	Nursing care for continuous renal replacement therapy
Raurell-Torredà et al. (20)	2020	Pubmed	Recommendation	Early mobilization of critically ill patients
Wang et al. (21)	2024	CNKI	Summary of Evidence	Evidence summary of early mobilization in ICU patients undergoing continuous renal replacement therapy

### Results of the quality assessment of the included studies

#### Quality assessment results of expert consensus and recommendations

Two expert consensus papers were included in this study (14, 15). For one of the papers, all the items were rated as “Yes.” For the other items, all the items were rated as “Yes” except for item 6, “Are the proposed views inconsistent with previous literature?” which was rated as “No.” As shown in Table 3. One recommendation was included in the study (20). All the items were rated as “Yes” except for item 6, “Are the proposed views inconsistent with previous literature?” which was rated as “No.”

**Table 3:** The quality evaluation results of the expert consensuses.

Item	Hodgson et al. (14)	Tang et al. (15)
1. Is the source of the opinion clearly identified?	Yes	Yes
2. Does the source of opinion have standing in the field of expertise?	Yes	Yes
3. Are the interests of the relevant population the central focus of the opinion?	Yes	Yes
4. Is the opinion’s basis in logic, experience, or underlying evidence clearly stated?	Yes	Yes
5. Is the argument or position presented logically?	Yes	Yes
6. Is there any incongruence with the available literature or evidence, and if so, is it logically defended?	Yes	No

### Results of the quality assessment of the group standards

This study included one group standard (19). All the items were rated as “Yes” except for item 6, “Are the proposed views inconsistent with previous literature?” which was rated as “Unclear.”

### Results of the quality assessment of the systematic reviews

Two systematic reviews were included in this study (16, 17). In Nydahl et al. (16), all items were rated “Yes” except for the item “Did the review authors report on the sources of funding for the studies included in the review?,” which was rated “No.” In Mayer et al. (17), the item “Did the review authors report on the sources of funding for the studies included in the review?” was rated “No,” and the items “If meta-analysis was performed, did the review authors use appropriate methods for statistical combination of results?” and “If meta-analysis was performed, did the review authors assess the potential impact of risk of bias in individual studies on the results of the meta-analysis or other evidence synthesis?” were rated “Not Applicable,” while all other items were rated “Yes.” As shown in Table 4.

**Table 4:** Results of quality evaluation of included systematic reviews (*n* = 2).

Item	Nydahl et al. (16)	Mayer et al. (17)
1. Did the research questions and inclusion criteria for the review include the components of PICO?	Yes	Yes
2. Did the report of the review contain an explicit statement that the review methods were established prior to the conduct of the review, and did the report justify any significant deviations from the protocol?	Yes	Yes
3. Did the review authors explain their selection of the study designs for inclusion in the review?	Yes	Yes
4. Did the review authors use a comprehensive literature search strategy?	Yes	Yes
5. Did the review authors perform study selection in duplicate?	Yes	Yes
6. Did the review authors perform data extraction in duplicate?	Yes	Yes
7. Did the review authors provide a list of excluded studies and justify the exclusions?	Yes	Yes
8. Did the review authors describe the included studies in adequate detail?	Yes	Yes
9. Did the review authors use a satisfactory technique for assessing the risk of bias (RoB) in individual studies included in the review?	Yes	Yes
10. Did the review authors report on the sources of funding for the studies included in the review?	No	No
11. If meta-analysis was performed, did the review authors use appropriate methods for statistical combination of results?	Yes	Not applicable
12. If meta-analysis was performed, did the review authors assess the potential impact of risk of bias in individual studies on the results of the meta-analysis or other evidence synthesis?	Yes	Not applicable
13. Did the review authors account for risk of bias in individual studies when interpreting/discussing the results of the review?	Yes	Yes
13. Did the review authors provide a satisfactory explanation for, and discussion of, any heterogeneity observed in the results of the review?	Yes	Yes
14. If they performed quantitative synthesis, did the review authors investigate publication bias and discuss its likely impact on the results of the review?	Yes	Yes
15. Did the review authors report any potential sources of conflict of interest, including any funding they received for conducting the review?	Yes	Yes

### Results of the quality assessment of quasiexperimental studies

This study included one quasiexperimental study (18). All the items were rated as “Yes” except for item 4, “Was a control group established?” which was rated as “No.”

### Quality assessment results of evidence summaries

By tracing back to the original studies, four guidelines, one expert consensus, and two systematic reviews were included. Among the four guidelines, three were rated as recommendation level A, and one was rated as level B. For expert consensus, item 6 was rated as “No,” whereas all other items were rated as “Yes.” For the two SRs, all the items were rated as “Yes.”

### Synthesis of best evidence

The included evidence was hierarchically classified according to the Joanna Briggs Institute (JBI) levels of evidence and grades of the recommendation system (2014 edition) (13). Ultimately, 20 items with the best evidence were synthesized and summarized across five domains. The details are presented in Table 5.

**Table 5:** Summary of best evidence on early rehabilitation for ICU patients undergoing continuous renal replacement therapy (CRRT).

Item	Evidence summary	Level of evidence
Multidisciplinary collaboration	1. The early rehabilitation management team should be composed of a multidisciplinary team, including rehabilitation therapists, medical staff, hemodialysis personnel, and ICU nursing staff. The attending physician will lead the team, with collaborative decision-making from all parties involved (14, 16, 18).	5
Preparation and assessment before activity	2. At least 2–4 healthcare professionals are required to collaborate in completing the task. This includes two bedside nurses, one physical therapist, and, if necessary, a respiratory therapist (18).	2
	3. Safety is a critical issue that must be prioritized before initiating early rehabilitation for patients (16).	1
	4. Before making decisions regarding active mobilization, the patient’s current condition should serve as the primary evaluation criterion. Additionally, the patient’s status and trends over the past few hours must be considered. Hemodynamic, respiratory, and cognitive status assessments should be conducted, alongside continuous cardiopulmonary monitoring and appropriate support equipment, to ensure patient safety (14, 16).	1
	5. Tools for assessing patient mobility and activity levels include muscle mass, strength, and physical function. These tools consist of muscle strength assessment (MRC scale), the Chelsea Critical Care Physical Assessment (CPAx), the Intensive Care Unit Physical Function Test (PFIT), and the ICU Mobility Scale (IMS) (20).	5
	6. Preparation of monitoring devices: Equip with a portable ventilator and monitor, suction device, oxygen supply, and a manual resuscitator (if IMS ≥ 7). Predict safety risks and prepare contingency plans. Assess airway pressure (20).	5
	7. Preparation of tubing: Before mobilization, check that all artificial airways (i.e., endotracheal tube, nasotracheal tube, or tracheostomy tube, CRRT catheter) are properly and securely placed. Ensure adequate tubing length for the planned activity, verify the position and type of CRRT catheter (flexible or rigid), assess the total length of the catheter, and ensure the stability of the patient-end port (17). Ensure that the CRRT device circuit connections are unobstructed (14, 16–18, 20).	2
	8. Hemodynamic stability in CRRT patients (Hemodynamic stability: Achieving 20% of baseline within 10 min post-activity; mean arterial pressure ≥ 65 mmHg). Low-dose catecholamines: Norepinephrine ≤ 0.1 mcg/kg/min (dosage may vary by patient population), with a stable infusion rate; the drug dose has not been increased in the past 5 h; infusion dose has been reduced. However, if CRRT is still required to maintain fluid balance or volume control, early mobilization may still be performed (18).	2
Activity contraindications	9. Relative contraindications: The use of vasopressors is not an absolute contraindication to mobilization; however, the appropriateness of mobilization is influenced by the absolute dosage and any changes in dosage (e.g., an increase in dosage should lead to caution or contraindication for mobilization) (14).	5
	10. Absolute contraindications: Unstable vital signs, kinking or twisting of the CRRT catheter, catheter disconnection, risk of catheter displacement, and inability to resolve CRRT machine alarms (18).	5
Exercise rehabilitation strategies	11. Timing of early mobilization: Assess the patient’s condition within 48 h of initiating CRRT treatment in ICU patients, and proceed with mobilization (18, 21). The assessment includes hemodynamic stability (referencing Evidence 8); respiratory parameters, including an inspired oxygen fraction ≤0.6, oxygen saturation ≥90%, and respiratory rate ≤30 breaths/min; a RASS score between-1and + 1; absence of CRRT alarms; and no relative or absolute contraindications (referencing evidence 9 and 10)	2
	12. Scope of early mobilization: Early mobilization for CRRT patients in the intensive care unit is safe and feasible both in bed and at the bedside. The functionality of the CRRT machine limits higher levels of activity, therefore, mobilization is restricted to activities near the bed or in proximity to the CRRT machine (17).	1
	13. Stage and Method of Early Mobilization: ① Baseline: Bed-based passive exercises, such as bridging exercises and upper limb training, etc. ② Stage 0: Introduction of passive and/or active range of motion (ROM) exercises, building upon the baseline; ③ Stage 1: Transition from swinging the lower limbs at the bedside to using a lift or other transfer devices to move the patient from the bed to a chair; ④ Stage 2: Bedside standing with assisted mobility (14, 18).	2
	14. Criteria for stopping activity: A change in systolic blood pressure (SBP) > 20% compared to baseline at rest; heart rate ≥200—age; a decrease in blood oxygen saturation of 5% compared to baseline at rest; Borg scale score ≥7 during exertion (0 = very easy, 10 = maximum effort). According to the Borg scale, a score ≥7 warrants an initial brief rest, followed by reassessment. If there is no improvement, activity should be stopped. If catheter displacement or hemodynamic instability occurs, activity must be immediately discontinued (14, 20, 21).	5
Monitoring during activity	15. Circuit management during activity: During patient mobilization, a designated person should monitor the CRRT circuit to identify and resolve any pressure alarm causes. If catheter displacement is detected, activity should be immediately stopped, and the catheter should be protected. In the case of catheter disconnection, local pressure should be applied, activity should be halted, and the legs should be elevated (17, 18).	2
	16. In the presence of a femoral catheter, hip flexion should not exceed 90° (20).	5
	17. Patients with internal jugular catheters may have a higher level of mobility; however, exercise rehabilitation for CRRT patients with femoral venous catheterization is also safe (20).	1
	18. Vital signs, CRRT treatment parameters, and fluid intake/output should be monitored and recorded every hour (19).	5
	19. Provide adequate enteral nutrition support in a timely manner to supply energy for early mobilization and rehabilitation exercises (15).	5
	20. Management of Common Adverse Events: During early rehabilitation exercises for CRRT patients, the two most common adverse events are hypotension requiring intravenous vasopressor treatment and transient disconnection of the CRRT catheter. Activity should be promptly stopped, and appropriate interventions should be initiated (17).	1

### Activity content at different stages

#### Flowchart of initiation criteria for early rehabilitation in ICU patients undergoing CRRT

**Figure 2:**
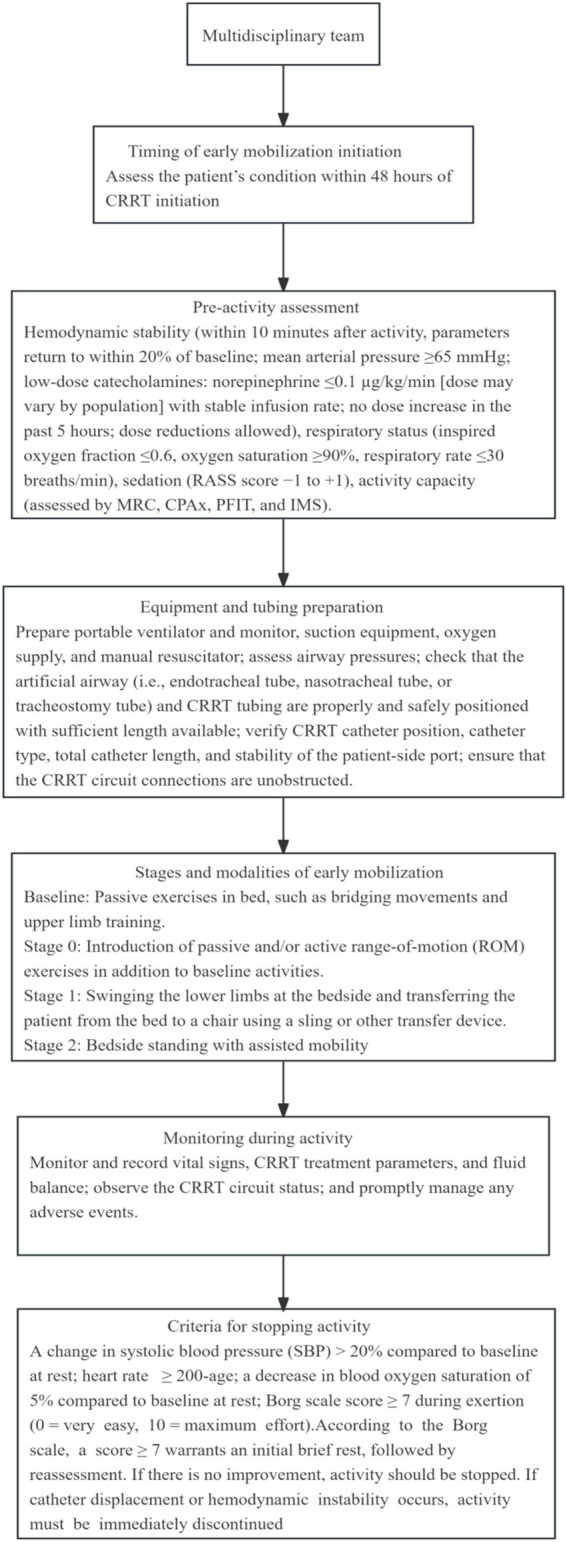
Flowchart of initiation criteria for early rehabilitation in ICU patients undergoing CRRT.

To better present the initiation process for early rehabilitation in ICU patients undergoing CRRT, we have summarized the following flowchart to provide a reference for clinical practice. As shown in Figure 2.

#### Clinical outcomes of patients reported in the included studies

Among the included studies, three reported clinical outcomes of the participants. The findings of Nydahl et al. (16) indicate that during rehabilitation activities, the incidence of adverse events was 2.6%, with the most frequently reported events being decreases in oxygen saturation and hemodynamic changes, accounting for 69% of adverse events, followed by intravascular catheter dislodgement or dysfunction, accounting for 65%. Mayer et al. (17) reported two major adverse events (hypotension requiring vasopressors and CRRT circuit disconnection, with a combined incidence of 0.24%) and 13 minor adverse events (combined incidence of 1.55%). The intervention improved patient outcomes, including enhanced muscle strength, shorter duration of mechanical ventilation, and improved quality of life at 2 months post-ICU discharge. No falls, dialysis access dislodgement, or other serious adverse events occurred during activity. Within 48 h of initiating CRRT, patients achieved varying degrees of early mobilization, with 88.5% able to perform passive and/or active activities, 9.2% able to transfer to a chair, and 1.8% able to ambulate with assistance, consistent with the findings reported by Talley et al. (18).

## Discussion

### Strengthening multidisciplinary team collaboration in early rehabilitation for ICU patients receiving continuous renal replacement therapy

Hodgson et al. (14) reported that expert consensus, systematic reviews (16), and quasiexperimental studies (18) emphasize the need to promote multidisciplinary team collaboration to make optimal early rehabilitation decisions. In Evidence 1, the expert consensus recommends that participation in early mobilization should be determined by all members of the multidisciplinary team, including rehabilitation therapists, medical staff, and nurses, with the clinical doctor bearing the final responsibility for the decision. The other two pieces of evidence also suggest the inclusion of hemodialysis personnel. Although there is currently no unified standard specifying which personnel should be involved in the early mobilization of patients, many studies have highlighted the importance of team collaboration. Multidisciplinary collaboration is a key factor and an important safeguard to ensure the effective implementation of early rehabilitation activities (22, 23). During rehabilitation exercises, it is essential to select experienced rehabilitation therapists with senior professional titles to provide hands-on therapy for the patient. Additionally, close cooperation between ICU nursing staff and hemodialysis personnel is needed to ensure patient safety and enhance rehabilitation outcomes. Furthermore, with strong domestic support for the development of intensive care subspecialty talent, critical care rehabilitation plays an important role in clinical diagnosis, treatment, and nursing (24). In the future, efforts should be made to actively explore the inclusion of critical care rehabilitation personnel in multidisciplinary collaboration for the early mobilization of patients receiving CRRT. Although the overall quality of this evidence is not high, its content provides significant benefits for both patients and healthcare professionals, and its adoption is still recommended.

### Fully improve preparation and assessment before activity to ensure patient safety

Prior to mobilization, CRRT patients should undergo thorough preparation, which not only enhances rehabilitation outcomes but also ensures patient safety. The areas that require preparation include personnel readiness, patient preparation, monitoring device setup, and tubing preparation. Evidence 2 suggests that two bedside nurses, one physical therapist, and, if necessary, a respiratory therapist should be present during patient mobilization. The evidence level is relatively high, and its adoption is recommended. Clear and rational personnel allocation ensures the effective execution of early mobilization, facilitating smooth progression and successful implementation of rehabilitation training (25). Through collaboration within a multidisciplinary team, responsibilities can be appropriately assigned—for example, one primary nurse monitors the patient’s vital signs, a rehabilitation therapist guides the exercise, and another nurse manages various lines and equipment. This arrangement allows for real-time intervention and risk management before, during, and after activity. Clear allocation of roles not only enhances the efficiency and standardization of activity implementation but also significantly reduces the risk of activity-related complications, thereby supporting the effectiveness and continuity of early rehabilitation. Evidence 4–5 suggests that, prior to mobilization, it is necessary to assess the patient’s circulatory, respiratory, and cognitive status, taking into account their condition and trends over the past few hours. The patient’s activity capacity and level should also be evaluated. Assessment tools varied across studies, reflecting differences in patients’ clinical conditions, functional levels, and the specific focus of rehabilitation goals. Regardless of the tool selected, its application should fully consider the patient’s individual condition—including disease severity, stability of vital signs, and cognitive and motor abilities—to ensure the accuracy and objectivity of the assessment results. A systematic review by Wang et al. (26) recommended the Chinese version of the CPAx scale as an early rehabilitation assessment tool for assessing physical function in adult ICU patients. However, this study acknowledges certain limitations and calls for further research to comprehensively validate its effectiveness. In addition, pre-activity monitoring and evaluation of all equipment are essential to ensure the smooth conduct of patient activities. Emergency measures should also be in place, including backup power in the event of an outage and availability of alternative devices in case of equipment failure. Line and tubing safety should be carefully monitored throughout the activity. Prior to mobilization, all lines must be properly secured, free from kinking, compression, or disconnection, and appropriately positioned. If catheter dislodgement occurs, the activity should be immediately halted (27), and the patient’s condition promptly assessed with appropriate interventions implemented.

### Accurate assessment of activity contraindications

Studies 9 and 10 have outlined the absolute and relative contraindications for patient mobilization. In some studies (18, 28, 29), contraindications to early mobilization have been defined, including objective criteria such as intracranial hypertension, unstable fractures, clinically unstable conditions (e.g., acute myocardial infarction), frequent unresolved CRRT alarms, a RASS score ≥2, an inspired oxygen fraction >0.6, positive end-expiratory pressure (PEEP) > 10 cmH₂O, and a mean arterial pressure <60 mmHg or >120 mmHg. The specific safety thresholds varied across studies. Furthermore, some studies have noted that there is currently insufficient evidence to provide definitive guidance on absolute activity contraindications (30). Therefore, patient contraindications should be assessed carefully and comprehensively, and early mobilization should be implemented collaboratively within a multidisciplinary team. However, evidence 9 and the expert consensus by Hodgson et al. (14) suggest that the use of vasopressors is not an absolute contraindication for activity, but dosage changes and their impact on the patient should be carefully considered. Therefore, in patients receiving vasoactive medications, early rehabilitation or out-of-bed mobilization should be preceded by a multidisciplinary assessment that systematically evaluates key indicators such as hemodynamic stability, level of consciousness, organ perfusion, and medication responsiveness, to ensure patient safety while maximizing potential benefits.

### Formulating a scientific early mobilization strategy

Early mobilization can improve physical function in CRRT patients (31). Most studies suggest (32, 33) that early mobilization in ICU patients should be initiated within 24–72 h of ICU admission. The evidence cited in this study (18, 21), indicating that ICU patients undergoing CRRT can be assessed and begin early mobilization within 48 h, falls within this recommended time frame. However, it should be noted that regardless of the timing, the prerequisite for initiating an early mobilization plan is the stability of the patient’s vital signs. Owing to the multiple tubes used in ICU patients and the large size of the CRRT machine, these factors significantly limit the patient’s range of motion. Therefore, it is best to restrict mobilization to the bedside or the vicinity of the machine to ensure activity safety (17). Guidelines and quasiexperimental studies also suggest that patient mobilization should follow a phased and progressive approach. At different stages, the focus of activity differs, with rehabilitation training progressing from low- to high-intensity levels (Table 6). In clinical practice, only a small number of patients are able to achieve high levels of activity intensity, and the implementation of early rehabilitation interventions is relatively rare (34, 35). These limitations are associated with various factors, including catheter displacement, hemodynamic instability, sedation, and supportive conditions. Therefore, early rehabilitation strategies for CRRT patients require further scientifically sound and feasible clinical practice for refinement and validation, with the ultimate goal of developing individualized and efficient rehabilitation plans. Furthermore, multiple pieces of evidence (14, 20, 21) have identified indicators for terminating activity. If changes in blood pressure, heart rate, oxygen saturation, or the Borg scale score occur or if emergencies such as catheter displacement or hemodynamic instability arise, activity should be immediately stopped. Safety must always be the primary consideration. A scientifically sound and feasible early rehabilitation plan is crucial for CRRT patients, as it can improve physiological function, accelerate recovery, and prevent complications. In recent years, the expanding use of specialized physical therapy modalities in ICU patients has drawn attention to their potential applicability in those undergoing CRRT. Neuromuscular electrical stimulation (NMES), for instance, induces passive muscle contractions by delivering external electrical currents that mimic neural impulses (36, 37). NMES is characterized by a favorable safety profile and good tolerability. For patients receiving CRRT whose vital signs are not yet fully stabilized and who are unable to engage in active mobilization, NMES may serve as a feasible initial modality for early rehabilitation. Muscle contractions induced by NMES can locally enhance microcirculation and may exert anti-inflammatory effects via activation of specific signaling pathways (38), potentially synergizing with the overall therapeutic effects of CRRT. Thus, NMES offers an early, safe, and feasible strategy to promote muscle function recovery in ICU patients undergoing CRRT. Future prospective studies are warranted to determine the optimal implementation in this population, including stimulation parameters, target sites, and timing.

**Table 6:** Activity content at different stages.

Phase	Activity level and content
Baseline	Bed-based passive exercises
Phase 0	Introduction of passive and/or active range of motion (ROM) exercises, building upon the baseline
Phase 1	Passively moved to the chair
Phase 2	Bedside standing with assisted mobility

### Strengthening safety monitoring during activity

Safety is the prerequisite and primary concern for rehabilitation exercises. Strengthening safety monitoring during patient mobilization is essential. Vital signs, treatment parameters, and other relevant data should be recorded by designated personnel every hour. When performing rehabilitation exercises in ICU patients undergoing CRRT, certain risk events may occur, such as circuit clotting, changes in transmembrane pressure, accidental catheter dislodgement, or effects of activity on extracorporeal blood flow. Balancing patient safety with mobilization is therefore crucial. Limb movement and position changes during activity may lead to significant changes in pre-pump pressure, potentially triggering clotting within the filter. Consequently, patients’ coagulation status should be assessed prior to activity, and anticoagulation strategies optimized. For example, in patients receiving heparin anticoagulation, the intensity may be appropriately increased within a safe range before activity. The filter appearance and pressure parameters (including arterial pressure and transmembrane pressure) should be closely monitored for signs of clotting. Sudden position changes or vigorous muscle contractions should be avoided. During early mobilization, the CRRT circuit and tubing must be closely monitored by dedicated staff members. If catheter displacement or disconnection occurs, activity should be immediately stopped, and the access route should be protected. Ensure that the catheter drainage port is positioned optimally during changes in patient position. It has been suggested (20) that for patients with a femoral venous catheter, hip flexion should not exceed 90 degrees. For patients with internal jugular vein catheters, excessive rotation of the head toward the opposite side should be avoided. Additionally, timely and adequate enteral nutrition support should be implemented to provide the necessary energy for early mobilization and rehabilitation exercises. Ensuring safety is a critical prerequisite for early rehabilitation exercises, and a multidisciplinary team should develop detailed emergency protocols to guarantee the safety of the mobilization process.

### Limitations

This study has certain limitations. Currently, the number of studies on early mobilization in ICU patients undergoing CRRT is limited, and high-quality evidence on exercise strategies, such as randomized controlled trials, is lacking. Moreover, the quality of the available literature is variable, and findings are often extrapolated from non-CRRT ICU populations, limiting their applicability.

## Conclusion

This study summarizes 20 key pieces of evidence for early rehabilitation in ICU patients undergoing continuous renal replacement therapy (CRRT), covering five areas: multidisciplinary collaboration, preparation and assessment before mobilization, activity contraindications, exercise rehabilitation strategies, and monitoring during mobilization. These findings provide a reference for ICU nursing staff in implementing early rehabilitation. However, the evidence extracted currently comes from a limited number of high-quality original studies, with the majority being reviews and expert consensus. Therefore, future studies could be based on patients’ clinical conditions and integrate the best available evidence to conduct randomized controlled trials and prospective studies, develop standardized early rehabilitation protocols for ICU patients undergoing CRRT, and clarify key assessment indicators (safety, functional recovery, CRRT duration, ICU length of stay, and quality of life), aiming to provide more precise and reliable scientific support for research and practice in related fields.

## Data Availability

The datasets presented in this study can be found in online repositories. The names of the repository/repositories and accession number(s) can be found in the article/supplementary material.
